# Iceberg rupture: A case of massive right atrial myxoma resulting in pulmonary embolism

**DOI:** 10.1016/j.ijscr.2024.110655

**Published:** 2024-11-26

**Authors:** Qing-Hua Zhang, Zi-Lin Xiong

**Affiliations:** Department of Cardiovascular Surgery, Second Affiliated Hospital of Harbin Medical University, Harbin Medical University, Harbin, China

**Keywords:** Atrial myxoma, Pulmonary embolism, Transesophageal echocardiogram

## Abstract

**Introduction and importance:**

This case highlights the importance of echocardiography in diagnosing pulmonary embolism and the need for careful timing of preoperative transesophageal echocardiogram in patients with potentially detachable intracardiac masses as a tumor detachment during TEE led to a life-threatening pulmonary embolism.

**Case presentation:**

A 30-year-old man with a history of treated pulmonary embolism had progressive dyspnea. Tests revealed a mass in the right atrium and a new pulmonary embolism. During preoperative TEE, a free-floating mass disappeared, suggesting embolization. Surgery was immediately initiated, and a myxoma was removed from the right atrium and pulmonary artery.

**Discussion:**

Right atrial myxoma is a rare cause of PE. Routine echocardiography is essential for suspected PE patients. The detachment during TEE may be related to the procedure, handling, or anesthesia. Preoperative TEE can be a useful supplementary diagnostic tool but requires careful consideration of timing.

**Conclusion:**

For pulmonary embolism diagnosis, searching for the cause is important and echocardiography is valuable. For patients with right atrial myxoma and pulmonary embolism, timely diagnosis and surgical resection are necessary, with attention to the timing of preoperative TEE examination.

## Highlights

• A rare case of a 30-year-old man with a history of treated pulmonary embolism presented with a right atrial myxoma, which led to a new pulmonary embolism.
• The detachment of the myxoma during preoperative transesophageal echocardiography (TEE) emphasized the importance of careful consideration of the timing of TEE for patients with potentially detachable intracardiac masses.
• The successful surgical resection of the myxoma from the right atrium and pulmonary artery, along with the repair of the septum, and the patient's recovery after cardiopulmonary bypass and extubation, highlighting the importance of timely diagnosis and treatment for such cases.

## 1 Introduction

Myxoma is the most common benign cardiac tumor, making up about 50 % of all benign cardiac tumors [1]. It usually occurs in the left atrium (LA) and rarely in the right atrium (RA). In rare instances when it occurs in the right atrium, it can lead to complications like pulmonary embolism (PE) [2]. In these cases, surgical removal of the atrial tumor and total extraction of the pulmonary emboli can help prevent recurrence.

## 2 Case report

A 30-year-old male patient, diagnosed with PE at a primary healthcare centre a year ago due to dyspnea, received anticoagulation treatment and the condition was well controlled. A week before admission, he experienced progressive dyspnea from a cold. Chest X-ray revealed localized pericardium and right pleura thickening. The ECG indicated WPW syndrome.

**Fig. 1:**
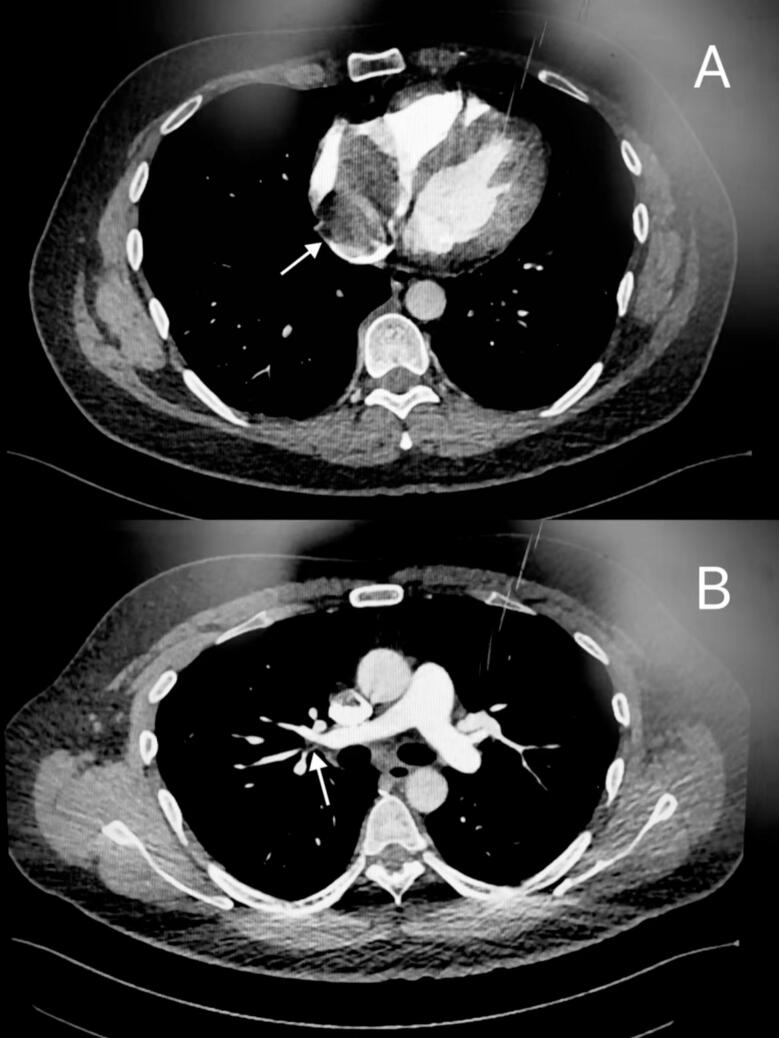
CT pulmonary angiography cross-section shows a massive irregular mass in the right atrium (A) and vascular embolism in the right lower lobe pulmonary artery branches (B).

After admission, the patient's CT pulmonary angiography revealed a calcified mass in the RA and a pulmonary embolism (Fig. 1). A solid mass was also found in the right atrium through echocardiography. Relevant laboratory tests and ultrasound examinations were performed to rule out other causes and surgical risks, all of which were largely normal. On the 4th day, the patient's symptoms worsened, leading to a scheduled surgery on the 5th day.

After general anesthesia in the operating room, preoperative transesophageal echocardiography (TEE) was performed to better evaluate the right atrial mass and the condition of the heart valves, and to assess the possibility of other cardiac masses or congenital septal (and other) defects that may have been missed during transthoracic echocardiography.

**Fig. 2:**
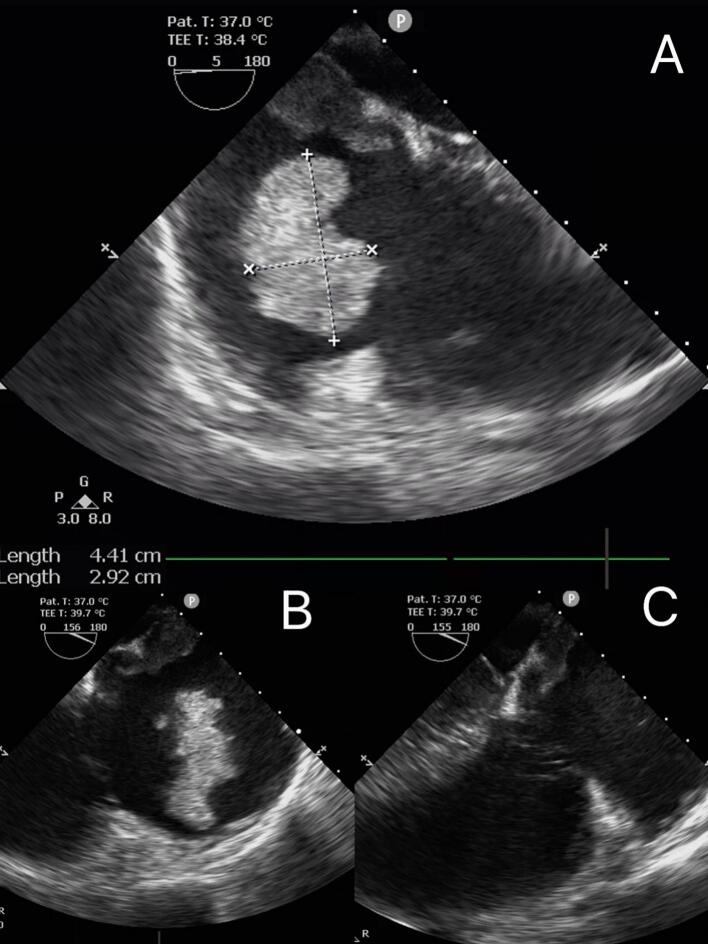
TEE shows a larger free-floating mass in the right atrium (A) TEE showing the mass in the right atrium (B) 5 min later, the same TEE view showed the disappearance of the mass within the right atrium (C).

TEE confirmed the presence of a mass in the right atrium, approximately 2.6 × 1.9 cm^2 in size, attached to the mid-septal region of the atrial septum. Another free, unattached irregular mass, approximately 5.8 × 2.8 cm^2 in size (Fig. 2A), was also observed in the right atrium. This mass was highly mobile, swinging with the blood flow towards the tricuspid valve, partially protruding into the right ventricle during diastole and retracting back into the right atrium during systole. At this point, the patient's hemodynamics were stable. When the TEE examination was nearing completion (Fig. 2B), the free-floating mass disappeared in the right atrium and right ventricle, suggesting embolization of the mass to the pulmonary artery (Fig. 2C). The right ventricle appeared dilated, and TEE showed significant tricuspid regurgitation. The patient experienced fluctuations in blood pressure and had difficulty maintaining circulation. Therefore, surgery was immediately initiated.

A midline sternotomy incision is performed, and extracorporeal circulation is established. The ascending aorta is clamped, and cardiac arrest is induced with Del Nido cardioplegia solution. A pedunculated tumor is visible within the RA, attached to the right atrial side of the atrial septum, measuring 2.0 × 3.0 cm. It appears yellow-green and mucoid, with lobes but no capsule, and is fragile. The tumor inside the right atrium is resected and removed, along with a 1.5 × 1.5 cm section of the atrial septum to which the tumor was attached. After confirming the absence of residual tumor, the atrial septum is sutured and repaired.

**Fig. 3:**
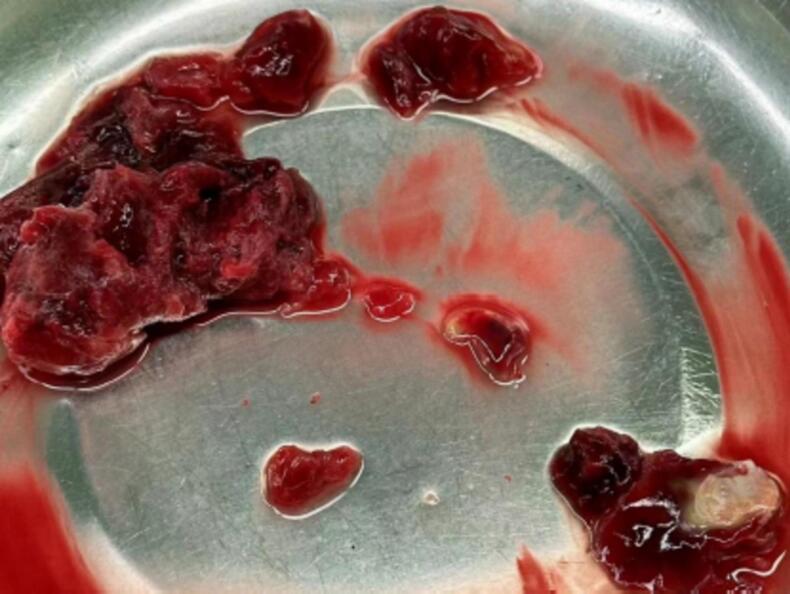
The gross appearance of the mucinous tumor.

There are no obvious masses found in the right ventricle or outflow tract upon exploration. The pulmonary artery is incised longitudinally, revealing a homogenous mass obstructing the main pulmonary artery at the bifurcation with a size of approximately 4.5 × 3.0 cm (Fig. 3). The mass is removed. The left and right pulmonary arteries are examined separately, and the mass is retrieved from the left lower pulmonary artery. Before closing the right atrium and pulmonary artery, repeated irrigation with warm saline is performed. There is no residual tumor, and the tricuspid valve is functioning well, with mild regurgitation and an area of 1 cm^2, as confirmed by TEE.

**Fig. 4:**
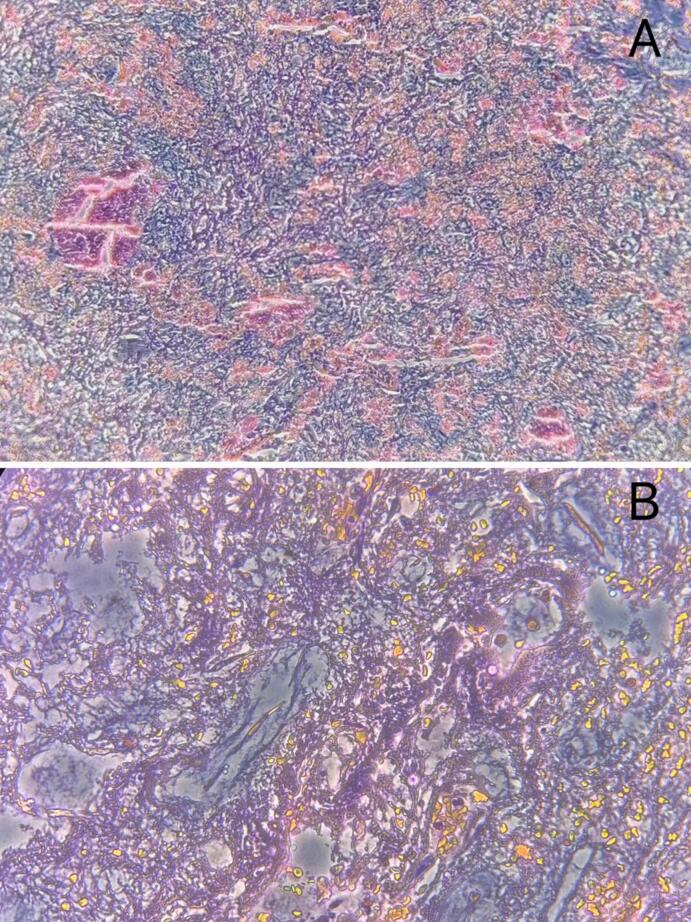
Under the microscope, loose mucous matrix can be seen surrounding the cells, with blood vessel formation in between. (A) Microscopic image at ×100. (B) Microscopic image at ×400.

The cardiopulmonary bypass lasts 65 min. The patient recovers and is extubated after 44 h, then leaves intensive care on day three. Histopathology confirms a myxoma diagnosis (Fig. 4).

## 3 Discussion

Right atrial myxoma, a rare cause of PE, was diagnosed in a patient receiving anticoagulant therapy for PE [1]. The diagnosis was confirmed through ultrasound when the patient had breathing difficulty a year later, as echocardiography wasn't performed initially due to stable hemodynamics.

In the diagnostic criteria for PE, echocardiography is only used as a means to exclude suspected PE with unstable hemodynamics and is not routinely used. Although right heart thrombus as a cause of pulmonary embolism is very rare, once the thrombus dislodges and causes acute PE, the patient may be at risk of a life-threatening condition, or even sudden death [4]. We believe that due to the lack of specific clinical manifestations of acute pulmonary embolism, and the important value of echocardiography in suggesting the etiological diagnosis of PTE and excluding other cardiovascular diseases, routine echocardiography is essential for patients suspected of PE.

The detachment of the right atrial myxoma occurred during the TEE procedure, which is temporally associated with the TEE procedure. However, there is no clear evidence to establish a causal relationship between the two. The handling and anesthesia prior to surgery may also contribute to the detachment of this large myxoma. Existing literature has reported the detachment of right atrial myxoma as a complication of TEE [3]. In this case, preoperative TEE was performed as a supplementary diagnostic tool to identify any cardiac abnormalities that might have been missed during transthoracic echocardiography, and it was performed before the start of the surgery. This effectively prevented the occurrence of life-threatening embolism caused by the detachment of the tumor, which would have been difficult to treat promptly. It also ensured a comprehensive evaluation of the cardiac tumor. Therefore, for potentially detachable intracardiac masses, it is advisable to avoid preoperative TEE examination or to perform the TEE examination before the start of the surgery. The work has been reported in line with the SCARE criteria [5].

## 4 Conclusion

For clear diagnosis of pulmonary embolism, it is still important to search for the cause, and echocardiography is of great value in diagnosis and exclusion. For patients with right atrial myxoma combined with pulmonary embolism, timely diagnosis and surgical resection should be performed, and attention should be paid to the timing of preoperative TEE examination.

## Informed consent

Written informed consent was obtained from the patient for the publication of this case report and its accompanying images. A copy of the consent form is available for review by the Editor-in-Chief upon request.

## Ethical approval

Ethical approval was not applicable for this study, as our Ethics Committee does not mandate approval for reporting individual cases or case series.

## Funding

This research did not receive any specific grants from funding agencies in the public, commercial, or not-for-profit sectors.

## Guarantor

Zi-Lin Xiong accept full responsibility for the work and/or the conduct of the study, had access to the data, and controlled the decision to publish.

## CRediT authorship contribution statement

Qing-Hua Zhang is the main conceiver of the thesis and is responsible for revising and proofreading the first draft of the paper.

Zi-Lin Xiong undertakes the collection of patient data and the writing of the first draft of the thesis.

## Declaration of competing interest

None.

## Data Availability

All relevant data are within the manuscript and its Supporting information files.
